# Long-Term Outcomes and Prognostic Analysis of Computed Tomography-Guided Radioactive ^125^I Seed Implantation for Locally Recurrent Rectal Cancer After External Beam Radiotherapy or Surgery

**DOI:** 10.3389/fonc.2020.540096

**Published:** 2021-01-21

**Authors:** Hao Wang, Lu Wang, Yuliang Jiang, Zhe Ji, Fuxin Guo, Ping Jiang, Xuemin Li, Yi Chen, Haitao Sun, Jinghong Fan, Gang Du, Junjie Wang

**Affiliations:** ^1^Department of Radiation Oncology, Peking University Third Hospital, Beijing, China; ^2^Department of Radiation Oncology, Bayannur Hospital, Bayannur, China

**Keywords:** locally recurrent rectal cancer, ^125^I seed implantation, dosimetry, prognosis, adverse effects

## Abstract

**Background:**

Management of locally recurrent rectal cancer (LRRC) after surgery or external beam radiotherapy (EBRT) remains a clinical challenge, given the limited treatment options and unsatisfactory outcomes. This study aimed to assess long-term outcomes of computed tomography (CT)-guided radioactive ^125^I seed implantation in patients with LRRC and associated prognostic factors.

**Methods:**

A total of 101 patients with LRRC treated with CT-guided ^125^I seed implantation from October 2003 to April 2019 were retrospectively studied. Treatment procedures involved preoperative planning design, ^125^I seed implantation, and postoperative dose evaluation. We evaluated the therapeutic efficacy, adverse effects, local control (LC) time, and overall survival (OS) time.

**Results:**

All the patients had previously undergone surgery or EBRT. The median age of patients was 59 (range, 31–81) years old. The median follow-up time was 20.5 (range, 0.89–125.8) months. The median LC and OS time were 10 (95% confidence interval (CI): 8.5–11.5) and 20.8 (95% CI: 18.7–22.9) months, respectively. The 1-, 2-, and 5-year LC rates were 44.2%, 20.7%, and 18.4%, respectively. The 1-, 2-, and 5-year OS rates were 73%, 31.4%, and 5%, respectively. Univariate analysis of LC suggested that when short-time tumor response achieved partial response (PR) or complete response (CR), or D_90_>129 Gy, or GTV ≤ 50 cm^3^, the LC significantly prolonged (P=0.044, 0.041, and <0.001, respectively). The multivariate analysis of LC indicated that the short-time tumor response was an independent factor influencing LC time (P<0.001). Besides, 8.9% (9/101) of the patients had adverse effects (≥grade 3): radiation-induced skin reaction (4/101), radiation-induced urinary reaction (1/101), fistula (2/101), and intestinal obstruction (2/101). The cumulative irradiation dose and the activity of a single seed were significantly correlated with adverse effects ≥grade 3 (P=0.047 and 0.035, respectively).

**Conclusion:**

CT-guided ^125^I seed implantation is a safe and effective salvage treatment for LRRC patients who previously underwent EBRT or surgery. D_90_ and GTV significantly influenced prognosis of such patients.

## Introduction

Locally recurrent rectal cancer (LRRC) refers to the recurrence, progression, or development of new sites within the pelvis after previous standard treatment for rectal cancer (1). Although preoperative chemoradiotherapy followed by total mesorectal excision (TME) significantly decreased the local recurrence rate, local recurrence has been reported in 5–11% of patients (2). Prognosis in LRRC patients is poor, with a median survival time of 10 months without treatment (3), and a reported 5-year survival rate of 10% (4). The majority of patients have severe symptoms such as pain, hematochezia, and fistula.

Surgery is an effective option and radical (R_0_) resection is an independent prognostic factor. Because the tumor typically shows extensive involvement in the pelvis, less than one-sixth of patients are eligible for R_0_ resection (5). The benefits of reirradiation include possible palliation by decreased steroid use, improvement in neurological symptoms, and extension of progression-free survival (PFS) and overall survival (OS) in some patients. Nevertheless, considering previous irradiation to the normal tissue, sufficient doses can hardly be delivered to the recurrent tumor in the pelvis (6). Furthermore, locally recurrent tumors are mostly located in the previously irradiated field, making it more challengeable for patients to undergo reirradiation (7). In addition, reirradiation with conventional radiation therapy confers a high rate of grade 3 adverse effects and late toxicities.

Nevertheless, ^125^I seed implantation can overcome the above-mentioned limitations. The dose of ^125^I seed is inversely proportional to the square of the distance, indicating that the dose is remarkably reduced surrounding the tumor. Interstitial implantation of ^125^I seeds delivers a high dose of radiation (140–180 Gy) to the tumor and spares surrounding normal tissues. In addition, ^125^I seed provides a slow continuous release of radiation that allows repair of sublethal damage and reoxygenation of hypoxic areas in the late-responding tissues. Therefore, radioactive ^125^I seed implantation might be a promising choice for the treatment of malignant tumors owing to its curative effect, minimal surgical trauma, and tolerable complications. The present study aimed to evaluate the efficacy and safety of computed tomography (CT)-guided ^125^I seed implantation for LRRC patients who underwent external beam radiotherapy (EBRT) or surgery, in addition to analysis of some prognostic factors.

## Patients and Methods

### Patients

This retrospective study collected the data of 101 patients with LRRC who were treated with CT-guided ^125^I seed implantation from October 2003 to April 2019. The study protocol was approved by the Ethics Committee of our hospital. All patients signed the written informed consent. The inclusion criteria were as follows: (1) patients with LRRC who were pathologically diagnosed; (2) extraluminal pelvic recurrence, without distal metastasis or with controllable oligometastasis; (3) tumor size < 7cm; (4) recurrence after surgery or EBRT, or refusal of surgery or EBRT; (5) life expectancy≥3 months. The patients’ median age was 59 (range, 31–81) years old. After tumor recurrence, all the patients received chemotherapy before seed implantation. And 17 (16.8%) patients received second-line or further chemotherapy. All the patients had received curative surgery or EBRT previously. Except for one case, 100 patients underwent surgery. Among all patients, 12 patients had no history of undergoing irradiation, 74 patients received one course of EBRT, and 14 patients received two courses of EBRT. The median cumulative dose in the pelvis was 50 (range, 30–130) Gy. All the patients had received chemotherapy previously. Demographic and clinical data of patients are listed in **Table 1**.

**Table 1:** Clinical details and patient demographics.

Characteristics	Value
Sex, n (%)	
Male	70 (69.3%)
Female	31 (30.7%)
Age (in years), range (median)	31-81 (59)
Histopathology	
Adenocarcinoma	100
Squamous carcinoma	1
Metastasis	
No metastasis, n (%)	82 (81.2%)
Distal metastasis, n (%)	19 (18.8%)
Lung	10 (10%)
Liver	7 (7%)
Prostate	1 (1%)
Axillary lymph nodes	1 (1%)
Previous surgery	
None	1
Once	86
Twice	13
Three times	1
Cumulative dose in the pelvis, EQD_2_ (Gy)	
<50	18
50-100	66
≥100	6
Unknown	11
Number of RT sessions	
0	12
1	74
2	14
3	1
GTV, mean ± SD (ml)	70.0 ± 45.3
Time from radiotherapy to seed implantation, (in months), range (median)	0.1–68.1 (17.0)

EQD_2_, equivalent dose in 2 Gy per fraction radiotherapy; RT, radiotherapy; GTV, gross tumor volume; SD, standard deviation.

### CT-Guided ^125^I Seed Implantation

Supine or prone position was chosen according to the tumor location. All the patients underwent contrast-enhanced CT scan with a slice thickness of 5 mm, one week before the implantation. CT data were transmitted to the brachytherapy treatment planning system (BTPS) (KLSIRPS-3D) which was provided by the Beijing University of Aeronautics and Astronautics and Beijing Astro Technology Co., Ltd. The radiation oncologists delineated the gross target volume (GTV) and organs at risk (OARs). Planned target volume (PTV) was defined as an extension of 5–10 mm from GTV. The optimal access for implantation (site, direction, and depth), prescription dose, number of seeds, single-seed activity, and seed distribution were designed.

**Figure 1:**
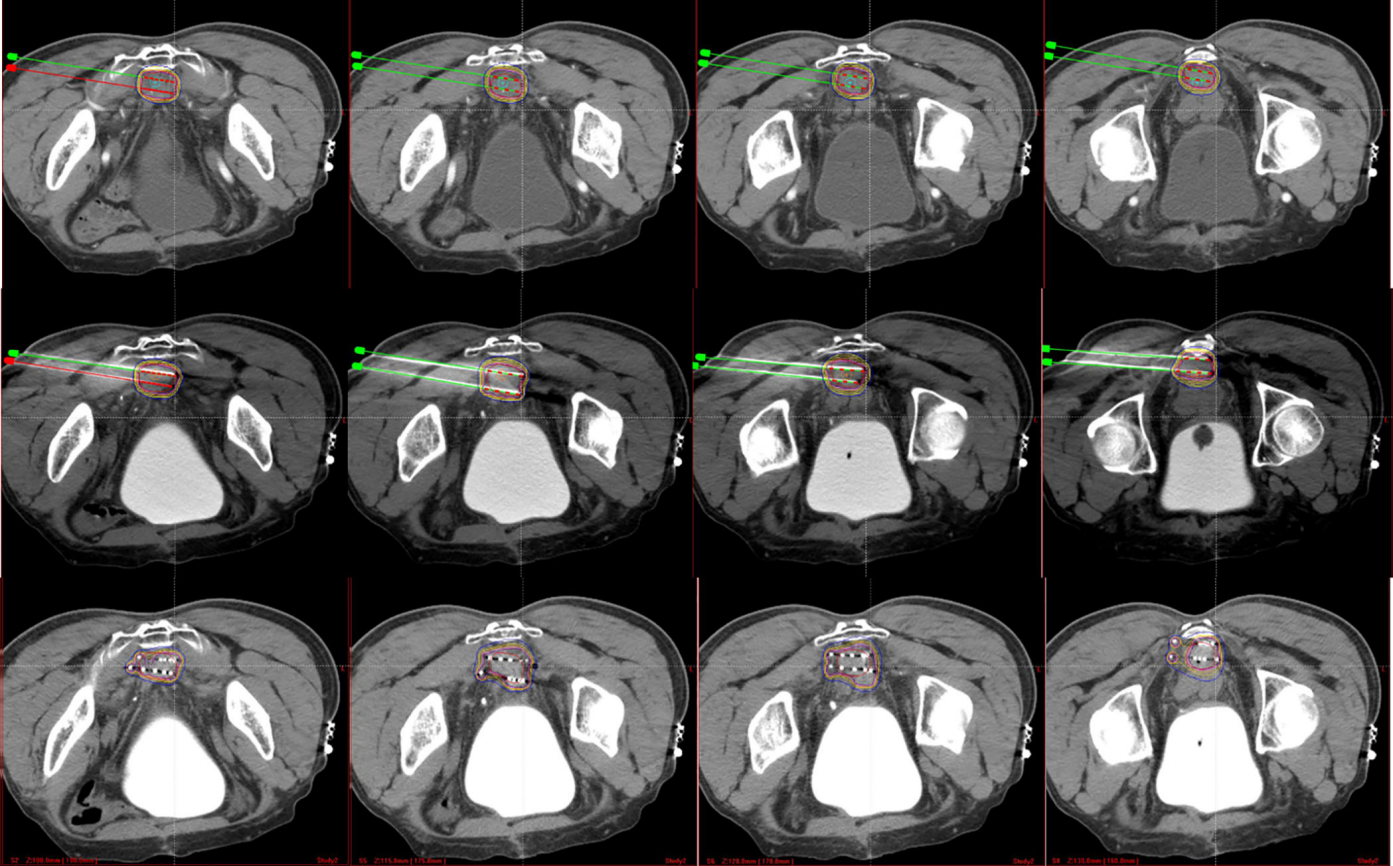
The first line presents the preoperative plan. The second line presents the intraoperative plan. The third line presents the postoperative plan.

Spinal anesthesia was induced in all patients. Under CT guidance, the needles were inserted into the planned site and arranged in parallel 5–10 mm apart. Then, the ^125^I seeds (6711_1985, Shanghai GMS Pharmaceutical Co., Ltd.) were implanted using a Mick seed implantation gun (Mick Radio-Nuclear Inc., Mount Vernon, NY, USA), by maintaining 1 cm between two seeds. After finishing seeds implantation, another CT scan was performed to verify the distribution of the seeds, and to calculate the dosimetric parameters (**Figure 1**). The postoperative parameters included: D_90_ (dose delivered to 90% of target volume), D_100_ (dose delivered to 100% of target volume), V_100_ (percentage of the target volume that was covered by 100% of the prescription dose), V_150_ (percentage of the target volume that was covered by 150% of the prescription dose), HI (homogeneity index), CI (conformal index), and EI (external index).

### Follow-Up

The patients were followed-up every 3 months by the radiation oncologists. The examinations included routine blood test, blood chemistry, tumor markers, magnetic resonance imaging (MRI) of pelvis, CT of the abdomen, and chest radiography. Positron emission tomography-CT (PET-CT) was employed when there were signs of metastasis. The local response was evaluated three months after ^125^I seed implantation by Response Evaluation Criteria in Solid Tumors (RECIST) version 1.1 (8). Complete response (CR) was defined as disappearance of all target lesions; partial response (PR) was defined as at least a 30% decrease in the diameters of the target lesion; progressive disease (PD) was defined as at least a 20% increase in the diameters of the target lesion; and stable disease (SD) was between PR and PD. The numeric rating scale (NRS) was used to assess the pain level. Adverse effects were evaluated according to the toxicity criteria of the Radiation Therapy Oncology Group (RTOG). Local control (LC) was defined as lack of tumor progression of the implanted volume.

### Statistical Analysis

All statistical analyses were carried out by SPSS 18.0 software (IBM, Armonk, NY, USA). LC and OS rates were calculated by plotting Kaplan–Meier curves. The log-rank test was employed for univariate analysis, and Cox proportional hazards regression model was used for multivariate analysis. The Chi-square test and Fisher’s exact test were undertaken to analyze factors correlated with adverse effects. The two-tailed P<0.05 was considered statistically significant. The curves were plotted with GraphPad Prism 5.0 software (GraphPad Software Inc., San Diego, CA, USA).

## Results

### Parameters of the Implantation

The volume of GTV was 6.5–234.8 (median, 66.9) cm^3^. The activity of a single radioactive seed was 0.4–0.8 (median, 0.66) mCi. The number of seeds was 6–137 (median, 70). The postoperative parameters included D_90_ (110.7 ± 33.7) Gy, D_100_ (46.8 ± 24.4) Gy, V_100_ (68.9 ± 36.4) %, V_150_ (56.8 ± 17.5) %, HI (0.34 ± 0.14), CI (0.91 ± 0.55), and EI (0.79 ± 1.6).

### Efficacy and Adverse Effects

The follow-up time was 0.89–125.8 (median, 20.5) months. The local response included 23 cases of CR, 35 cases of PR, 33 cases of SD, and 10 cases of PD. The objective response rate (ORR) was 57.4% (58/101). Adverse effects occurred in 14 (13.9%) patients, including 21 cases. Besides, 12 (57.1%) of the cases showed grades 1–2 adverse effects, including neuropathy (n=1, 4.8%), radiation-induced skin reaction (n=4, 19%), and radiation-induced urinary reaction (n=7, 33.3%). Additionally, 9 (42.9%) cases had adverse effects with ≥grade 3, including radiation-induced urinary reaction (n=1, 4.8%), fistula (n=2, 9.5%), intestinal obstruction (n=2, 9.5%), and radiation-induced skin reaction (n=4, 19.1%). Concerning implantation-related complications, seed migration was observed in two patients during the follow-up, and one patient developed needle-tract implantation metastases. No correlation was found between D_90_ (D_90_ ≤ 129 Gy vs. D_90_>129 Gy) and adverse effects ≥grade 3 (P=0.160) (**Table 2**). The cumulative irradiation dose (≤100 Gy vs. >100 Gy) and the activity of a single seed (≤0.68 mCi vs. >0.68 mCi) were significantly correlated with adverse effects ≥grade 3 (P=0.047 and 0.035, respectively). The rates of adverse effects (grade ≥3) for cumulative dose ≤100 Gy and >100 Gy were 5.9% and 40%, respectively, and the rates of adverse effects (grade ≥3) for the activity of a single seed ≤0.68 mCi and >0.68 mCi were 3.4% and 16.3%, respectively.

**Table 2:** Analysis of factors associated with adverse effects.

Factors	Adverse effects		Total (n)	P
	Grade 0–2	≥Grade 3		
D_90_ (Gy)				
≤129	63 (94.0%)	4 (6.0%)	67	0.160
>129	29 (85.3%)	5 (14.7%)	34	
Total (n)	92	9	101	
Cumulative dose in the pelvis, EQD_2_ (Gy)				
≤100	80 (94.1%)	5 (5.9%)	85	0.047
>100	3 (60.0%)	2 (40.0%)	5	
Total (n)	83	7	101	
Activity of a single seed (mCi)				
≤0.68	56 (96.6%)	2 (3.4%)	58	0.035
>0.68	36 (83.7%)	7 (16.3%)	43	
Total (n)	92	9	101	

D_90_, dose that covers 90% target volume; EQD_2_, equivalent dose in 2 Gy per fraction radiotherapy.

### Local Control

**Figure 2:**
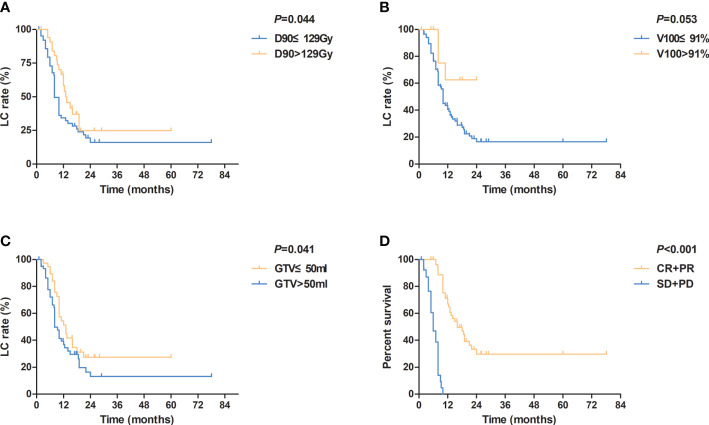
Kaplan-Meier curves for local control according to **(A)** different values of D_90_ (≤ 129Gy vs. >129Gy); **(B)** different values of V_100_ (≤ 91% vs. >91%); **(C)** different values of GTV (≤ 50 ml vs. >50 ml); **(D)** different tumor responses (CR+PR vs. SD+PD). D_90_, dose that covers 90% target volume; V_100_, percentage of the target volume that was covered by 100% of the prescription dose; GTV, gross tumor volume; CR, complete response; PR, partial response; SD, stable disease; PD, progressive disease.

The median LC time was 10 (95% confidence interval (CI): 8.5–11.5) months. The 1-, 2-, and 5-year LC rates were 44.2%, 20.7%, and 18.4%, respectively. The univariate analysis of LC showed that D_90_ (≤129 Gy vs. >129 Gy), GTV (≤50 cm^3^ vs. >50 cm^3^), and short-time tumor response (CR+PR vs. SD+PD) significantly influenced LC time (P=0.044, 0.041, and <0.001, respectively) (**Table 3**). Besides, a prolonged trend was shown in LC when V_100_>91% (P=0.053) (**Figure 2**). The 1-year LC rate for V_100_ ≤ 91% and V_100_>91% was 42.1% and 62.5%, respectively. Multivariate analysis of these factors influencing LC time indicated that short-time tumor response was an independent factor of LC time (hazard ratio [HR] = 0.072; 95% CI=0.034–0.153; P<0.001). The LC of CR and PR was superior to that of SD and PD. The median LC time for (CR+PR) and (SD+PD) was 16.0 and 6.0 months, respectively.

**Table 3:** Univariate and multivariate analysis of factors influencing local control.

Factors		n	Median (months)	Univariate analysis	Multivariate analysis		
				P	HR	95% CI	P
Age (in years)	≤40	8	12	0.668			
	41-65	63	10				
	>66	30	10				
D_90_	≤129 Gy	67	8	0.044			
	>129 Gy	34	13				
V_100_	≤91%	89	10	0.053			
	>91%	12	–				
Activity of a single seed	≤0.68 mCi	58	10				
	>0.68 mCi	43	10	0.587			
GTV	≤50 cm3	38	13	0.041			
	>50 cm3	63	8				
Cumulative dose in the pelvis (EQD_2_)	≤100Gy	85	10	0.765			
	>100Gy	5	12				
Tumor response	CR+PR	58	16	<0.001	0.072	0.034–0.153	<0.001
	SD+PD	43	6				

HR, hazard ratio; CI, confidence interval; D_90_, dose that covers 90% target volume; V_100_, percentage of the target volume that was covered by 100% of the prescription dose; GTV, gross tumor volume; EQD_2_, equivalent dose in 2 Gy per fraction radiotherapy; CR, complete response; PR, partial response; SD, stable disease; PD, progressive disease.

### Overall Survival

The median OS time was 20.8 (95% CI: 18.7–22.9) months. The 1-, 2-, and 5-year OS rates were 73%, 31.4%, and 5%, respectively. In the current research, 93 patients died at the end of the follow-up. Of these, 20 patients died of local recurrence, 54 died of metastasis, seven died of non-tumor causes, and 12 died of unknown causes. For the univariate analysis, only the short-time tumor response was significantly correlated with OS time (P=0.017). The median OS time for (CR+PR) and (SD+PD) was 22.0 and 14.8 months, respectively.

## Discussion

Numerous therapeutic modalities have been used for patients with LRRC, including surgery, EBRT, intraoperative radiotherapy (IORT), high-dose-rate (HDR) brachytherapy, chemotherapy, etc. Previously irradiated patients were found less sensitive to chemotherapy than those who did not receive pelvic radiotherapy (6, 9). Furthermore, chemotherapy alone is not effective for controlling pelvic recurrence. It was reported that the 5-year survival rate for R_0_ resection ranged from 43% to 60% (10). However, only a limited number of patients were eligible for R_0_ resection. LRRC typically presents with extensive involvement of the tumor in the pelvis, making it a great challenge to perform resection. Moreover, distorted anatomical structures and tissue fibrosis from previous irradiation increase the difficulty in surgery (6, 11). Besides, extensive resection is typically followed by high morbidity and mortality risks (10).

Several scholars have pointed out that re-irradiation is a reasonable option for patients with LRRC who have undergone EBRT previously. Besides, it could relieve symptoms to some extent (11–13). A systematic review reported 375 patients with LRRC who were reirradiated. Reirradiation was mostly administered using hyperfractionated or 1.8 Gy once-daily chemoradiotherapy. Median survival time was 39–60 months for resected patients and 12–16 months for palliative patients. The symptomatic relief rate was 82%–100% (12). Nevertheless, it is challenging to deliver a definitive dose to the tumor lesions considering history of irradiation to the normal pelvic tissue (6). Late toxicity rates were high. Recently, with the advances of cutting-edge technologies, a more precise radiotherapy technique, namely stereotactic ablative radiotherapy (SABR), was used to treat LRRC. Murray et al. (14) performed a systematic review regarding the use of SABR for the reirradiation of recurrent malignant disease within the pelvis, to guide the clinical implementation of this technique, and demonstrated that for previously irradiated patients with recurrent pelvic disease, SABR re-irradiation could be a feasible intervention for those who otherwise have limited options. Dagoglu et al. (15) reported the outcomes of a series of patients with pelvic recurrences from colorectal cancer reirradiated with SBRT. They employed Cyberknife Robotic Stereotactic Radiosurgery system with fiducial based real time tracking, and noted that one patient had small bowel perforation and required surgery (grade IV), two patients had symptomatic neuropathy (grade III), and one patient developed hydronephrosis from ureteric fibrosis requiring a stent (grade III).

IORT refers to the direct irradiation of the tumor surgically. A number of scholars have used IORT alone in the treatment of patients with history of undergoing irradiation. However, their results were not satisfactory. The LC and OS rates of IORT alone were significantly lower than those of IORT combined with EBRT (13, 16, 17). Besides, a significant increase was observed in the rate of complications related to the wound and neuropathy.

Several studies reported HDR brachytherapy for the management of LRRC. HDR intraluminal brachytherapy plays a great therapeutic role in the treatment of intraluminal tumor recurrence. And HDR interstitial brachytherapy has also shown an impressive therapeutic efficacy. Sakurai et al. (18) reported that LC was achieved in 7 of 18 patients with LRRC at a median follow-up time of 14.4 months. Morimoto et al. (19) studied 9 patients, and it was demonstrated that the 8-year OS, LC, and PFS rates were 56%, 44%, and 33%, respectively. Three patients had grade 3 adverse effects. Nevertheless, it should be noted that the tumor location of these patients could be reached by a needle applicator through the perineum. Lateral or presacral recurrence was contraindicated for HDR brachytherapy, which, however, could be managed with ^125^I seed implantation.

Recurrent tumor after EBRT or surgery is typically associated with a poor blood supply. Permanent implantation of ^125^I seeds has significant advantages in killing hypoxic tumor cells by consistently radiating low-dose rays. Furthermore, it also has the major advantages of delivering a high dose of irradiation to the tumor with a very sharp fall-off outside the implanted volume. For patients who are not eligible candidates for reirradiation, surgery or HDR interstitial brachytherapy, ^125^I seed implantation might be an alternative treatment option.

The results of the current research were comparable to those reported previously related to application of ^125^I seed implantation for LRRC. In our study, the median LC time was 10 months, and the median OS time was 20.8 months. The first report related to application of brachytherapy for LRRC included 30 patients (20). The seeds were implanted after radical or debulking surgical resection. The LC rate was 37.5% for gross residual disease and 66% for microscopic residual disease. The tumors in 64% (18/30) of patients were still under control at the last follow-up. No mortality was observed, and the morbidity rate was low. Martinez et al. (21) reported 29 patients with recurrent colorectal cancer in the pelvis or the paraaortic lymph nodes treated with intraoperative ^125^I seed implantation. The implanted volume received a median minimal peripheral dose of 140 Gy to total decay. The 1-, 2-, and 4-year LC rates were 38%, 17%, and 17%, (median, 11 months), respectively. The 1-, 2-, and 4-year OS rates were 70%, 35%, and 21%, (median, 18 months), respectively. Overall, 45% (13/29) of patients experienced 15 adverse events. Image-guided percutaneous ^125^I seed implantation which was minimally invasive has gradually become the mainstream treatment approach. In Wang et al.’s study (22), 15 patients with LRRC received ^125^I or ^103^Pd seed implantation under CT guidance. The median minimal peripheral dose was 150 Gy. The median follow-up, LC, and OS time were 8, 7, and 9 months, respectively. Only one patient had a grade 4 toxic event. Wang et al. (23) reported 20 patients with LRRC who were treated with CT-guided ^125^I seed implantation. The median peripheral dose was 120 Gy. CR or PR was achieved in 75% of patients. The median survival time was 18.8 months. The 1- and 2-year survival rates were 75% and 25%, respectively. Nevertheless, none of the above-mentioned studies analyzed optimal parameters and factors related to adverse effects. The optimal dosimetric parameters for ^125^I seed implantation are still elusive except for prostate cancer. In prostate cancer, the prognosis of patients with D_90_≥140 Gy was significantly greater than those with D_90_<140 Gy. The outcomes of patients with V_100_≥90% were also markedly superior than those with V_100_<90% (24–26). Similarly, for LRRC, the present study revealed that patients with D_90_>129 Gy achieved a notably longer LC time than those with D_90_ ≤ 129 Gy. The median LC time for D_90_>129 Gy and D_90_ ≤ 129 Gy was 8 and 13 months, respectively. Moreover, a trend of prolonged LC time was observed in patients with V_100_>91%. The 1-year LC rate for V_100_ ≤ 91% and V_100_>91% was 42.1% and 62.5%, respectively.

Regarding adverse effects, a meta-analysis of irradiation for LRRC showed that the rates of adverse effects (≥grade 3) for acute and late complications were 11.7% and 25.2%, respectively (12). Bhangu et al. (27) summarized surgical outcomes of 22 studies on LRRC and revealed that the overall rate of complications was 51%. In the current research, the overall rate of adverse effects was 13.9% (14/101), and 8.9% (9/101) of patients had ≥grade 3 adverse effects. The complication rates reported in our study were relatively lower than those reported in studies that used other treatment modalities. Cumulative irradiation dose (≤100 Gy vs. >100 Gy) and the activity of a single seed (≤0.68 mCi vs. >0.68 mCi) were found to be correlated with adverse effects (≥grade 3). We considered that the adverse effects in 2 patients with cumulative irradiation dose >100 Gy might be attributed to the late complications of previous high-dose irradiation. When low-activity seeds are used, the influence of a single seed on dosimetry is reduced, leading to a better dose homogeneity. The misplacement of a single seed would cause less damage to surrounding normal tissue. Sloboda et al. (28) reported that a range of 0.4–0.6 mCi per seed was optimal to cover the target volume and spare the urethra in prostate cancer. However, in the present study, the activity of a single seed had no effect on either LC or OS.

There are a number of limitations in this study. First, considering the short half-life of ^125^I seed, all the dosimetric parameters were postoperative parameters which were calculated immediately after ^125^I seed implantation, with assumption of complete dose delivery. According to the physical characteristic of ^125^I seed, 65% of prescription dose was delivered in 3 months and 90% was delivered in about 6 months. All the patients were still alive 3 months after ^125^I seed implantation except for one patient who died of pulmonary infection one month after the implantation. Moreover, the majority of patients were still alive 6 months after ^125^I seed implantation. Thus, it could be concluded that the postoperative dosimetry was nearly close to the delivered dosimetry. Nevertheless, there may still exist some minor errors that require further investigation. Second, the treatment modalities used for LRRC patients before ^125^I seed implantation were not consistent (e.g., some patients did not receive irradiation), which might influence patients’ sensitivity to ^125^I seed implantation and clinical outcomes. In addition, treatment modalities used for LRRC patients after ^125^I seed implantation were not consistent as well. A number of patients received postoperative chemotherapy, while others poorly tolerated, which might lead to the low efficiency of ^125^I seed implantation on OS. Third, it was sometimes difficult to indicate whether the adverse effects were caused by ^125^I seed implantation, tumor progression, or previous high-dose irradiation. In such cases, we attributed the adverse effects to ^125^I seed implantation, which might lead to an overestimation of the rates of adverse effects. Last but not the least, this was a single-center retrospective study with small sample size.

In conclusion, CT-guided ^125^I seed implantation is a safe, effective, and minimally invasive treatment for LRRC patients with mild adverse effects. This treatment does not require patients to have a high physical strength and is not limited by previous irradiation dose. Patients with LRRC after previous EBRT with limited treatment options are especially proper candidates for ^125^I seed implantation. Nevertheless, multicenter studies with a larger sample size and prospective design are needed to further investigate the effects of ^125^I seed implantation on LRRC patients.

## Data Availability Statement

The datasets generated for this study are available on request to the corresponding author.

## Ethics Statement

The studies involving human participants were reviewed and approved by the Institutional Review Boards of Peking University Third Hospital (IRB00006761). The patients/participants provided their written informed consent to participate in this study.

## Author Contributions

LW, HW, HS, and GD were responsible for the collection of the clinical data. LW and HW drafted the manuscript. JW was in charge of verifying the patients’ implantation plan and directing the writing. YJ, ZJ, FG, PJ, XL, YC, and JF were responsible for the supplement and refinement of the clinical data and collectively carried out the implantation plan. HS was responsible for the design and production of radioactive seed implantation plan. All authors read and approved the final manuscript. LW and HW contributed equally to the article and are co-first authors of the article. All authors contributed to the article and approved the submitted version.

## Conflict of Interest

The authors declare that the research was conducted in the absence of any commercial or financial relationships that could be construed as a potential conflict of interest.
